# Differenzialdiagnostische Unterscheidung zwischen substanzinduzierten und primären Psychosen: Empfehlungen für die allgemeinpsychiatrische und forensische Praxis

**DOI:** 10.1007/s00115-021-01083-3

**Published:** 2021-03-03

**Authors:** Dusan Hirjak, Andreas Meyer-Lindenberg, Geva A. Brandt, Harald Dreßing

**Affiliations:** Klinik für Psychiatrie und Psychotherapie, Zentralinstitut für Seelische Gesundheit, Medizinische Fakultät Mannheim, Universität Heidelberg, 68159 Mannheim, Deutschland

**Keywords:** Substanzinduzierte Psychose, Primäre Psychose, Differentialdiagnose, Forensische Psychiatrie, Klinische Symptome, Substance-induced psychosis, Primary psychosis, Differential diagnosis, Forensic psychiatry, Clinical symptoms

## Abstract

Substanzinduzierte psychotische Störungen (SIPS) sind häufig und für ca. 25 % der ersten Einweisungen in eine psychiatrische Klinik verantwortlich. Aus klinischer Sicht ist aufgrund ähnlicher psychopathologischer Phänomene die diagnostische Unterscheidung zwischen SIPS und primären (genuinen oder kryptogenen) psychotischen Störungen oft eine Herausforderung. Dieser Umstand wird dadurch erschwert, dass SIPS im Zusammenhang mit Cannabis, Halluzinogenen und Amphetaminen ein erhebliches Risiko des Übergangs in eine primäre psychotische Störung (z. B. Schizophrenie) haben. Im ersten Abschnitt dieser Arbeit werden zunächst zwei exemplarische Fallvignetten aus der allgemeinpsychiatrischen und forensischen Praxis vorgestellt. Danach wird im Sinne einer selektiven Literaturübersicht die Relevanz der differenzialdiagnostischen Unterscheidung beider Störungsbilder aus der Sicht der allgemeinen und forensischen Psychiatrie in Bezug auf Therapie, Prognose und richterliche Entscheidung bezüglich der Unterbringung im Maßregelvollzug (§ 63 vs. § 64 StGB) beleuchtet. Der letzte Abschnitt hat das Ziel, ein strukturiertes Vorgehen zur differenzialdiagnostischen Unterscheidung zwischen SIPS und primären psychotischen Störungen zu erarbeiten. Die in dieser Arbeit dargestellten und diskutierten Konzepte und Befunde sollen klinisch tätigen Psychiatern und Psychologen die Diagnosestellung im allgemeinen und forensischen Kontext erleichtern.

## Abstract (en)

Substance-induced psychotic disorders (SIPD) are frequent and account for about 25% of the first admissions to a psychiatric hospital. From a clinical point of view the differential diagnosis of SIPD vs. primary (genuine or cryptogenic) psychotic disorders is often a challenge due to the similar psychopathology. This is complicated by the fact that SIPDs associated with cannabis, hallucinogens and amphetamines have a significant risk of transition to manifest psychotic disorder (e.g. schizophrenia). In the first section of this paper two case reports from general psychiatric and forensic practice are presented. Then, in a narrative review the relevance of the differential diagnostic distinction between both disorders is examined from the perspective of general and forensic psychiatry with respect to therapy, prognosis and judicial decisions regarding the placement in forensic commitment (§ 63 vs. § 64 German Penal Code, StGB). The last section aims to develop a structured procedure for the differentiation between SIPD and primary psychotic disorders. The concepts and findings presented and discussed in this paper are intended to help psychiatrists and psychologists make a diagnosis in a general and a forensic context.

## Hintergrund

In der allgemeinpsychiatrischen Praxis ist die Unterscheidung zwischen einer substanzinduzierten psychotischen Störung (SIPS) und einer primären (genuinen oder kryptogenen) psychotischen Störung (z. B. Schizophrenie, schizoaffektive Störung, schizophreniforme Störung, akut-polymorph psychotische Störung), welche oft mit dem Konsum psychotomimetischer Substanzen einhergeht, entscheidend für das Verständnis des Krankheitsverlaufs (inklusive Prognose) und die Planung einer angemessenen psychiatrischen Behandlung (v. a. Dauer der antipsychotischen Behandlung), insbesondere wenn die psychotische Störung erst kürzlich aufgetreten ist [1]. Substanzen mit psychotomimetischen Eigenschaften sind weit verbreitet, und ihr Konsum oder Missbrauch kann bei Menschen, die ansonsten frei von schweren psychischen Erkrankungen sind, akute psychotische Symptome hervorrufen, die unter Umständen eine stationäre psychiatrische Krisenintervention erfordern [2, 3]. Die akuten Symptome einer SIPS sind bei einem Großteil der Konsumenten vorübergehender Natur und durch Störungen des formalen und inhaltlichen Denkens, Wahrnehmungsstörungen (z. B. Halluzinationen), Wutausbrüche, Selbstverletzungen (ggf. Suizidversuche), Aggressionen und gelegentlich auch körperlichen Übergriffen charakterisiert. Aus epidemiologischer Sicht handelt sich deshalb um – in der klinischen Praxis – häufige Störungen. Die Inzidenz von SIPS reicht von 1,5 bis 6,5 Personen pro 100.000 Einwohner, ähnlich wie bei den geschätzten Inzidenzraten für affektive Erkrankungen (4,6–6,1 Episoden pro 100.000; [4]). Bis zu 25 % der ersten Krankenhauseinweisungen wegen Psychosen können die Diagnose einer SIPS enthalten [3]. In Hochrisikopopulationen, wie z. B. bei Menschen mit häufigem Cannabis- oder Amphetaminkonsum, liegt die Prävalenz von SIPS bei 20–40 % [2, 5]. Bei einem signifikanten Anteil der Patienten kann die SIPS wiederkehren und trotz adäquater Behandlung andauern bzw. in eine manifeste Schizophrenie übergehen und oft mit einer Vielzahl negativer Folgen verbunden sein [6]. Die spezifischen Übergangsraten waren in den bisherigen Studien sehr unterschiedlich (11,3 % in der Studie von Kendler et al. [7], 17,3 % in Schottland [8] und 46 % in Finnland [9]). Ein Großteil dieser Varianz dürfte auf Unterschiede in der Nachbeobachtungszeit und der Definitionsbreite zurückzuführen sein [7]. Die Rate des Substanzkonsums bei Menschen mit schweren psychischen Erkrankungen ist höher als die der Allgemeinbevölkerung [10], u. a. auch bei Erstmanifestation einer Psychose [11].

In der wissenschaftlichen Literatur werden 4 unterschiedliche Erklärungsmodelle, warum Patienten mit primären psychotischen Störungen eine höhere Prävalenz von Substanzmissbrauch als die Normalbevölkerung haben, diskutiert ([12–14]; für Übersicht s. Meister et al. [15]): (1) Die sog. „Selbsttherapie- oder Selbstmedikationshypothese“ geht davon aus, dass die Patienten versuchen, Krankheitssymptome oder unerwünschte Nebenwirkungen der Antipsychotika zu lindern [16]. (2) Die Vertreter des Affektregulationsmodells postulieren, dass gerade der negative Affekt schizophrener Patienten zum Substanzmissbrauch prädisponiert [17], d. h. Patienten konsumieren illegale Substanzen (z. B. Cannabis) hauptsächlich zur Stimmungsaufhellung, insbesondere im Zusammenhang mit einer durch die Erkrankung verursachten Anhedonie und Antriebsreduktion, um unangenehme Zustände, Symptome und Langweile zu lindern [18, 19]. (3) Das Supersensitivitätsmodell besagt, dass Patienten mit primären Psychosen nach Substanzkonsum häufiger eine erneute psychotische Exazerbation (aufgrund der neurobiologischen Überempfindlichkeit bzw. „Supersensitivität“) und seltener eine körperliche Substanzabhängigkeit entwickeln als Patienten mit einer manifesten Suchterkrankung [20, 21]. Bisher konnte dieses Modell nicht bestätigt werden. (4) Die Social-drift-Hypothese postuliert einen besseren Zugang zu psychotropen (oft illegalen) Substanzen durch den sozioökonomischen Abstieg und Wohnsitz in sozioökonomisch benachteiligten Stadtvierteln [22].

In der forensischen Praxis ist die Unterscheidung zwischen einer SIPS und einer primären psychotischen Störung ebenfalls relevant [23], weil sie die Entscheidungsgrundlage für die Art und Dauer der Unterbringung des nicht oder vermindert schuldfähigen Straftäters im Maßregelvollzug (§ 63 vs. § 64 des Strafgesetzbuches [StGB]) darstellt. Während die Unterbringung nach § 64 StGB zeitlich begrenzt (zumeist auf 2 Jahre) ist, ist die Unterbringung nach § 63 StGB nicht zeitlich befristet, sondern hängt von der klinischen Symptomatik und der Krankheitsprognose des jeweiligen Patienten ab.

Trotz der allgemeinpsychiatrischen und forensischen Bedeutung dieses Themas sind die Unterschiede zwischen SIPS und primären psychotischen Störungen im Hinblick auf die Pathogenese, Klinik und Prognose wenig untersucht worden [24]. Es ist immer noch umstritten, ob SIPS eine von der Schizophrenie getrennte Entität ist und ob die Diagnose über die Zeit stabil ist [25]. Einige Autoren argumentieren, dass es keine konsistenten Unterschiede in der Symptomatik von SIPS und primären psychotischen Störungen gibt [26, 27]. Andere Autoren behaupten, dass trotz phänomenologischer Ähnlichkeiten die Möglichkeit einer nosologisch eindeutigen Diagnose bestehen bleibt [28]. Eingebettet in der aktuellen Datenlage soll in dieser Arbeit der nosologische und diagnostische Stellenwert der SIPS im allgemeinpsychiatrischen und forensischen Kontext dargestellt werden, um Fehler bei der Diagnosestellung und der anschließenden Therapie sowohl im allgemeinpsychiatrischen als auch im forensischen Kontext zu vermeiden.

Der erste Abschnitt basiert auf zwei typischen Fallvignetten aus der allgemeinpsychiatrischen und forensischen Praxis. Der zweite Abschnitt ist dem neurobiologischen, klinischen und differenzialdiagnostischen Hintergrund der SIPS im allgemeinpsychiatrischen und forensischem Kontext gewidmet und fasst im Sinne einer selektiven Literaturübersicht die Ergebnisse rezenter Studien und Übersichtsarbeiten zu diesem Thema zusammen. Im letzten Abschnitt werden innovative Fragestellungen, die gutachtliche Anwendbarkeit und algorithmenbasierte Handlungsempfehlungen für die differenzialdiagnostische Unterscheidung zwischen SIPS und primären psychotischen Störungen vor dem Hintergrund aktueller Studien präsentiert und diskutiert.

## Untersuchungsmethoden

Diese Arbeit bedient sich zweier Untersuchungsmethoden: (1) Um dieses Thema konkreter und anschaulicher zu machen, werden im ersten Abschnitt zwei Fallvignetten aus der allgemeinpsychiatrischen und forensischen Praxis vorgestellt und analysiert, wobei besonders auf differenzialdiagnostische Überlegungen aufmerksam gemacht wird. Bei der forensischen Kasuistik handelt es sich um einen juristischen Text. Die Zielsetzung ist es, in enger Rückbindung an die selbst erhobenen Daten und an die Patientenakten, die Fallstricke in der Diagnosestellung bzw. der forensischen Begutachtung zu beleuchten. (2) Für den zweiten Abschnitt dieser Arbeit wurde bis zum 31.10.2020 eine selektive Literaturrecherche in PubMed durchgeführt, wobei die Suchbegriffe „substance induced psychosis“, „drug induced psychosis“, „psychosis“, „schizophrenia“, „schizophrenia spectrum disorders“, „differential diagnosis“, „violence“, „homicide“ oder deren Kombinationen und die Bereiche, die sich mit der differentialdiagnostischen Abgrenzung von SIPS und primären psychotischen Störungen beschäftigen, verwendet wurden. Die in dieser selektiven Literaturübersicht („narratives Review“) enthaltenen Artikel wurden nicht auf systematischer Basis ausgewählt. Die identifizierten Artikel wurden anschließend im Konsens zwischen DH und HD diskutiert. Trotz unserer umfangreichen Suche in PubMed und den verwendeten Suchbegriffen können wir nicht ausschließen, dass einige relevante Publikationen/Studien übersehen wurden.

## Allgemeinpsychiatrische Fallvignette

Der 27-jährige Patient Herr T. wurde im Oktober 2019 wegen erneuter akuter Exazerbation einer seit 2010 bekannten paranoiden Schizophrenie über die Notfallambulanz des Zentralinstituts für Seelische Gesundheit (ZI) in Mannheim stationär aufgenommen. Er war zum Aufnahmezeitpunkt arbeitssuchend und berichtete, dass er unter Todesängsten leide, keinen klaren Gedanken fassen könne, vieles auf sich beziehe und „die Musik“ zu ihm spreche. Er habe zu Beginn des Jahres einen schweren Verkehrsunfall erlitten und lebe seitdem wieder bei seiner Mutter. Er rauche seit dem Unfall regelmäßig Cannabis, um seine Schmerzen besser ertragen zu können. Vor dem Unfall habe er häufig auf Partys Amphetamine, Kokain und Ecstasy konsumiert. Seine Medikation (Quetiapin 800 mg/Tag) habe er nur 3 Monate nach dem ersten stationären Aufenthalt 2010 eingenommen und danach eigenständig abgesetzt.

### Psychiatrische Vorgeschichte

Nach dem Konsum von Cannabis und Amphetaminen sei es 2010 zur Exazerbation der Psychose gekommen, daraufhin erfolgte die erste stationäre Behandlung im ZI Mannheim mit der Diagnose paranoide Schizophrenie gemäß der International Classification of Diseases (ICD-10), aufgrund – nach Aussage der Behandler – einer deutlich „über die Intoxikation hinausgehenden psychotischen Symptomatik“. Nach 6‑monatiger Behandlung wurde Herr T. vollremittiert nach Hause entlassen. Herr S. beendete 2012 die Schule mit der Fachhochschulreife und absolvierte eine Ausbildung zum Industriemechaniker. In diesem Beruf arbeitete Herr T. bis kurz vor dem Verkehrsunfall 2019. Eigenanamnestisch kam es in den Jahren ohne Klinikaufenthalte zu mehreren kurzen vorübergehenden psychotischen Zuständen, stets im Zusammenhang mit dem Konsum illegaler psychotroper Substanzen. In diesen Phasen habe Herr T. visuelle und akustische Halluzinationen, formalgedankliche Defizite im Sinne von Gedankenrasen, Gedankenabrissen und Ideenflucht sowie inhaltliche Denkstörungen im Sinne von Verfolgungswahn, Beziehungswahn, und Größenwahn erlebt.

### Epikrise

Bei der Aufnahme 2019 standen folgende Symptome im Vordergrund: Formalgedankliche Defizite, psychomotorische Unruhe, paranoides Beeinträchtigungserleben, akustische Halluzinationen und bizarrer Wahn. Im stationären Verlauf kam es zu einer Zunahme der psychomotorischen Unruhe bis hin zu aggressiven Durchbrüchen, sodass bei akuten und erheblichen Gefährdungsaspekten eine zeitweilige Unterbringung nach Psychisch-Kranken-Hilfe-Gesetz (PsychKHG) Baden-Württemberg notwendig war. Herr T. wurde im Verlauf mit Haloperidol 10 mg/Tag, bei gutem Ansprechen im Jahre 2010 und unzureichender initialer Wirkung von Quetiapin, Olanzapin und Risperidon, unter zusätzlicher Gabe von Diazepam (max. Dosis 40 mg/Tag) behandelt. Im weiteren Verlauf konnte auf eine Monotherapie mit Quetiapin (600 mg/Tag) umgestellt werden. Die Medikation mit Diazepam konnte komplikationslos ausgeschlichen werden. In der kognitiven Testung nach Teilremission der zur Aufnahme führenden Symptomatik mittels Brief Cognitive Assessment Tool for Schizophrenia (B‑CATS; [29]) erreichte Herr T. stets Werte, welche oberhalb der Werte von Patienten mit Schizophrenie, schizoaffektiver Störung und schizophreniformer Störung lagen (Trail Making Test [TMT]-B: 62s, verbale Flüssigkeit RW: 39, Zahlen-Symbol-Test [ZST] RW: 73, WP = 10; [30]). Herr T. konnte im Februar 2020 in teilremittiertem Zustand (Positive and Negative Syndrome Scale, PANSS [31] 10/2019 P-Score: 32, N‑Score: 19, G‑Score: 55; PANSS 02/2020 P-Score: 15, N‑Score: 14, G‑Score: 41) entlassen werden und erschien regelmäßig zur ambulanten Weiterbehandlung in der Track-Einheit [32] am ZI. Im ambulanten Setting konnte eine durchgehende Abstinenz von illegalen Substanzen erreicht werden. Die Medikation mit Quetiapin (600 mg/Tag) wurde zunächst beibehalten und der Patient nahm regelmäßig am ambulanten verhaltenstherapeutisch orientierten Entwöhnungsprogramm CANDIS („cannabis use disorder“; [33, 34]) teil. Unter der multimodalen Therapie kam es im weiteren Verlauf zur Vollremission der psychotischen Symptomatik (PANSS 11/2020 P-Skala: 8, N‑Skala: 8, G‑Skala: 18) sowie zu einer weiteren Stabilisierung kognitiver Funktionen (B-CATS 11/2020 TMT B: 41s, ZST RW: 76, WP = 11, verbale Flüssigkeit RW: 37). In Zusammenschau der erhobenen Befunde und kritischer Würdigung des Gesamtverlaufs (vor allem der fehlenden Abstinenz bei Erstmanifestation im Jahre 2010) wurde die initiale Diagnose der paranoiden Schizophrenie in eine substanzinduzierte psychotische Störung gemäß ICD-10 umgeändert.

## Forensische Fallvignette (BGH 5 StR 540/04 – Urteil vom 01.02.2005, LG Berlin)^1^

Der zur Tatzeit 30-jährige Beschuldigte konsumiert etwa seit 1997 Drogen, vornehmlich in der „Technoszene“ gebräuchliche Psychostimulanzien wie LSD, Speed, Ecstasy und Amphetamine. Seit 1999 entwickelte er nach Einnahme von Drogen verstärkt mit optischen und akustischen Halluzinationen einhergehende massive Angstzustände. Im Sommer 2000 hatte er sich Feuerwehrleuten widersetzt, die wegen eines derartigen akuten Zustandes von seiner Mutter alarmiert worden waren, und hatte, nach einer Verfolgungsfahrt gestellt, im Zustand der Schuldunfähigkeit auf zwei Polizeibeamte mit einer einem Beamten entrissenen Dienstwaffe mit Tötungsvorsatz geschossen. Die deshalb gegen den Beschuldigten angeordnete Unterbringung in einer Entziehungsanstalt war nach knapp 10-monatigem Maßregelvollzug mit Rücksicht auf die Verweigerungshaltung des Beschuldigten, der sich ausschließlich einer Therapie außerhalb des Maßregelvollzugs stellen wollte, für erledigt erklärt worden. Schon während des Maßregelvollzugs hatte der Beschuldigte einen Drogenrückfall erlitten, nach dem er wiederum paranoid wurde und eine behandelnde Ärztin verletzte. Auch in der anschließenden externen Drogentherapie hatte er alsbald einen Rückfall erlitten. Nach deren Beendigung im November 2001 war er rund eineinhalb Jahre drogenfrei geblieben. Seit Frühjahr 2003 konsumierte er wieder Ecstasy und später gesteigert Speed und Amphetamine. Nach verstärktem Ecstasy- und Amphetaminmissbrauch litt der Beschuldigte an Kreislaufproblemen; er bekam zudem wiederum optische und akustische Halluzinationen, dabei fühlte er sich und seine Familie bedroht. Seine Mutter, zu der er sich in diesem akuten Angstzustand begab, erkannte er im Rahmen der Psychose alsbald nicht mehr. Er bewaffnete sich mit zwei Messern mit jeweils rund 15 cm Klingenlänge. Am Mittag des Folgetages alarmierte schließlich die Mutter des Beschuldigten, die selbst wegen seines bedrohlichen Verhaltens aus ihrer Wohnung geflüchtet war – die Großmutter hatte sich im Badezimmer verbarrikadiert –, die Polizei. Der Beschuldigte war offensichtlich „nicht Herr seiner Sinne“; er vermochte zustandsbedingt auch die Polizeibeamten als solche nicht wahrzunehmen. Nur mit großen Mühen und erheblichem Einsatzaufwand konnte er schließlich überwältigt werden. Zuvor lief er zweimal mit gezogenen Messern auf zwei Polizeibeamte zu und versuchte, freilich vergeblich, sie mit kraftvoll und wuchtig geführten Stichen ober- und unterhalb ihrer Schutzschilde zu treffen und zu verletzen (…). Dabei war möglicherweise bereits seine Unrechtseinsichtsfähigkeit, jedenfalls seine Steuerungsfähigkeit zustandsbedingt aufgehoben. Die Annahme des Landgerichts, dass in dem genannten Problembereich die Voraussetzungen des § 63 StGB vorliegen, ist ausreichend begründet und aus Rechtsgründen nicht zu beanstanden. An einem aufgrund der Beurteilung des Beschuldigten durch den psychiatrischen Sachverständigen festgestellten, zur Tatzeit zweifelsfrei gegebenen Zustand der Schuldunfähigkeit aufgrund eines stabilen geistigen Defekts fehlte es nicht. Ursache war, neben der diagnostizierten Betäubungsmittelsucht, eine zum wiederholten Male aufgetretene massive psychotische (Über‑)Reaktion des Beschuldigten von einiger Dauer auf eingenommene Suchtmittel. Diese ist bei psychiatrischer Diagnose einer substanzinduzierten psychotischen Störung (im Sinne von Diagnostic and Statistical Manual of Mental Disorders(DSM)-IV 292.11 bzw. 12, ICD-10 F 15.51 bzw. 52) – vom Landgericht rechtsfehlerfrei, ungefähr vergleichbar mit einer Alkoholüberempfindlichkeit, als krankhafte seelische Störung gewertet worden. Die für die Anwendung des § 63 StGB erforderliche Dauerhaftigkeit der Störung wird durch deren wiederholtes Auftreten nach immer wieder geübtem Betäubungsmittelmissbrauch, namentlich – wie hier – aufgrund einer Betäubungsmittelsucht, begründet (...).

## Ergebnisse und Diskussion

Auf Grundlage der Fallanalyse und der selektiven Literaturübersicht werden insbesondere die folgenden Themengebiete diskutiert: (1) klinische, neurobiologische und prognostische Charakteristika der SIPS und ihre differenzialdiagnostische Abgrenzung von primären psychotischen Störungen, (2) Fallstricke in der Diagnosestellung und forensischen Begutachtung und (3) algorithmenbasierte Handlungsempfehlungen für die differenzialdiagnostische Unterscheidung zwischen SIPS und primären psychotischen Störungen.

### Klinische Merkmale der SIPS und ihre differenzialdiagnostische Abgrenzung von primären psychotischen Störungen

SIPS sind kurze psychotische Syndrome, die durch den Substanzkonsum ausgelöst werden und noch Tage oder Wochen nach Abklingen der Substanzintoxikation fortbestehen [3]. Eine kürzlich diskutierte neue diagnostische Kategorie, die sog. substanzbezogene exogene Psychose („substance related exogenous psychosis“, SREP), schließt dabei sowohl die transiente als auch die persistierende Psychose im Zusammenhang mit Substanzkonsum ein [35]. Die SIPS sind gemäß der ICD-10-Klassifikation (F1x.5) als Psychosen, welche während oder nach dem Substanzgebrauch auftreten, definiert. Aus klinischer Sicht ist diese diagnostische Gruppe durch akustische und optische (selten gustatorische) Halluzinationen, Wahrnehmungsstörungen, paranoide (und bizarre) Wahnideen, Verfolgungswahn, Größenwahn (s. auch allgemeinpsychiatrische Fallvignette), psychomotorische Auffälligkeiten (Hyperkinesien, Stereotypien, Erregung oder Stupor) und Affektstörungen charakterisiert [36]. Nach DSM‑5 liegt eine SIPS vor, wenn die folgenden Kriterien erfüllt sind: (1) Vorliegen intensiver Halluzinationen oder Wahnvorstellungen; (2) Halluzinationen oder Wahnvorstellungen manifestieren sich während oder kurz nach dem Konsum einer Substanz oder eines Medikamentes, von denen bekannt ist, dass sie psychotische Symptome verursachen können; (3) psychotische Symptome sind nicht durch eine primäre (genuine) psychotische Störung (wie z. B. Schizophrenie, schizophreniforme Störung, schizoaffektive Störung oder akut-polymorph psychotische Störung) begründbar (d. h., wenn das psychotische Symptom vor dem Substanz- oder Medikamentenkonsum begonnen hat oder länger als einen Monat nach der Substanzeinnahme oder dem -entzug anhält, dann ist eine andere psychotische Störung wahrscheinlich); (4) psychotische Symptome dürfen nicht durch ein Delir begründbar sein. Sowohl bei der Diagnosestellung in der allgemeinpsychiatrischen Praxis als auch in der forensischen Beurteilung ist zu beachten, dass das Zeitkriterium für eine SIPS in der ICD-10- und DSM-5-Klassifikation unterschiedlich ist. Nach ICD-10 müssen sich die psychotischen Symptome kurz nach dem Substanzkonsum manifestieren und dürfen bis zu 6 Monaten nach dem letzten Substanzkonsum persistieren. Im DSM‑5 ist bei einem Symptombeginn innerhalb eines Monats nach Drogenkonsum die SIPS Diagnose möglich. In der DSM-5-Klassifikation darf die psychotische Symptomatik bei einer SIPS allerdings nicht länger als 4 Wochen anhalten.

Anhand der aktuellen Evidenz lässt sich sagen, dass die bei den primären psychotischen Störungen auftretenden Denkstörungen, welche durch eine Aufspaltung und Auflockerung von Assoziationen, eine Konkretisierung des abstrakten Denkens und eine Beeinträchtigung des zielgerichteten Denkens gekennzeichnet sind, bei SIPS weniger ausgeprägt sind [37, 38]. Eine Unterscheidung der beiden Krankheitsentitäten ausschließlich anhand der akuten Symptome ist jedoch nicht möglich [38, 39]. Im Vergleich zu primären psychotischen Störungen scheinen die SIPS eine schnellere Remission der psychotischen Symptomatik zu zeigen [40] und sistieren mit der Abstinenz, obwohl die Remission der psychopathologischen und kognitiven (visuell-räumliche Wahrnehmung, Exekutivfunktion und Abstraktion) Symptome unvollständig sein kann [38, 39, 41, 42].

Aus pathogenetischer Sicht können sich SIPS aus einer anhaltenden Exposition gegenüber psychotomimetischen Substanzen entwickeln (vor allem bei psychisch gesunden Personen) oder aus einer Mischung aus Drogenexposition und individueller Anfälligkeit für Psychosen entstehen. Darüber hinaus kann regelmäßiger Drogenkonsum, bei einer starken genetischen Vulnerabilität (Schizotaxie), eine primäre psychotische Störung auslösen [7]. Unabhängig von der Pathophysiologie sind gerade Patienten mit psychotischen Erkrankungen, die regelmäßig Substanzen konsumieren, eine komplexe Gruppe von Individuen mit vielen verschiedenen Bedürfnissen. Schwere psychische Erkrankungen und komorbider Substanzmissbrauch sind mit einer Reihe negativer Folgen wie reduzierter Adhärenz, häufigen Rückfällen und häufigeren Krankenhausaufenthalten verbunden [43]. In der groß angelegten Studie von Caton et al. [1] konnten signifikante Unterschiede in folgenden demographischen, familiären und klinischen Bereichen festgestellt werden: (1) elterlicher Substanzmissbrauch (stärker in der Gruppe der SIPS), (2) ein höherer Schweregrad psychopathologischer Symptome (stärker in der Gruppe der primären Psychosen), (3) eine gleichzeitige Diagnose von Drogenabhängigkeit (häufiger in der Gruppe der SIPS) und (4) visuelle Halluzinationen (häufiger in der Gruppe der SIPS). Eine aktuelle Übersicht [44] zeigte lediglich minimale Unterschiede in den demographischen und klinischen Merkmalen von Patienten mit SIPS im Vergleich zu anderen psychotischen Störungen. Patienten mit SIPS wiesen mit größerer Wahrscheinlichkeit eine komorbide Suchterkrankung auf. Die Autoren konnten allerdings keinen Unterschied in Bezug auf die Schwere der psychotischen Symptomatik, die Remissions- und Rückfallraten und die Länge der unbehandelten Psychose identifizieren [44]. Der einzige Unterschied zwischen den Gruppen bestand darin, dass die SIPS-Gruppe in Bezug auf den beruflichen Status schlechter abschnitt, wobei dieser Unterschied am ehesten durch die Suchterkrankung zu erklären ist [44]. Die diagnostische Unterscheidung ist besonders in den frühen Stadien einer psychotischen Störung von Bedeutung [45], da jede Störung eine andere Behandlung erfordert und Personen mit SIPS anfälliger für die unerwünschten Wirkungen antipsychotischer Medikamente sein können [7]. Im Vergleich zu Patienten mit einer primären psychotischen Störung benötigen Patienten mit SIPS nicht zwingend eine dauerhafte antipsychotische Medikation. Nach der aktuellen S3-Leitlinie Methamphetamin-bezogene Störungen [46] sind bei Methamphetamin-induzierter psychotischer Symptomatik atypische Antipsychotika (z. B. Olanzapin oder Quetiapin [47]) und ggf. zeitlich begrenzt begleitend Benzodiazepine empfohlen. Eine Überprüfung der Indikation und möglichst ein Absetzversuch sollten bei Vollremission der psychotischen Symptomatik und nach spätestens 6 Monaten erfolgen.

### Fallstricke in der forensischen Begutachtung

Psychotomimetische Substanzen können bei gesunden Personen und Patienten mit psychotischen Störungen relativ schwerwiegende Auswirkungen haben und zu einer Verschlimmerung der Symptome führen [44]. Sowohl SIPS als auch primäre psychotische Störungen können deshalb mit paranoiden Ängsten, Misstrauen, Gefühlen der akuten Bedrohung, Wutausbrüchen, Selbstverletzungen, Suizidversuchen, Aggressionen und Übergriffen einhergehen [44]. Dem Zusammenhang zwischen Gewalttaten, Tötungsdelikten und primären psychotischen Störungen wird seit Jahren große klinische, wissenschaftliche und nicht zuletzt auch mediale Aufmerksamkeit geschenkt [48]. Aktuelle große populationsweite und diagnoseübergreifende Studien haben gezeigt, dass das Tötungsrisiko bei Personen mit Psychosen 10- bis 20-mal höher als in der Allgemeinbevölkerung [49–53] ist (für eine Übersicht s. auch Maier et al. [48], Witt et al. [54] und Rund et al. [53, 55]). Darüber hinaus konnte auch ein moderater, jedoch konsistenter Zusammenhang zwischen gewalttätigem Verhalten und Schizophrenie nachgewiesen werden [54, 56]. Die Metaanalyse von Nielssen und Kollegen [57] an 10 Studien (894 Personen) konnte zeigen, dass 38,5 % der Tötungsdelikte während der Erstmanifestation einer psychotischen Erkrankung auftraten. Besonders interessant ist auch, dass die schwersten Formen der Gewalt mit Substanzmissbrauch assoziiert waren [58, 59]. Nielssen und Kollegen [60] fanden, dass 73 % der Patienten mit psychotischen Störungen, welche des Mordes beschuldigt wurden, vorher Substanzmissbrauch berichtet haben. Allerdings waren nur 35 % der Patienten auch zum Tatzeitpunkt intoxikiert [60]. Anhand der aktuellen wissenschaftlichen Evidenz lässt sich deshalb sagen, dass bei Menschen mit psychotischen Störungen (v. a. mit Schizophrenie) ein höheres Risiko für zwischenmenschliche Gewaltentwicklung (aller Arten) besteht [48].

Für die gutachtliche Beurteilung der Schuldfähigkeit gemäß §§ 20, 21 StGB ist die Differenzialdiagnose zwischen einer Schizophrenie (F20) und einer SIPS (F1x.5) von nachgeordneter Bedeutung. Eine für den Tatzeitpunkt festgestellte produktiv-psychotische Symptomatik mit z. B. Wahnsymptomatik, Wahrnehmungsstörungen i. S. von Halluzinationen, formalen Denkstörungen und affektiven sowie psychomotorischen Symptomen würde unabhängig von den oben diskutieren differenzialdiagnostischen Überlegungen unter das Eingangsmerkmal der „krankhaften seelischen Störung“ der §§ 20, 21 StGB subsumiert. Gutachtlich muss dann in einem nächsten Schritt geprüft werden, ob zwischen der psychopathologischen Symptomatik und der rechtswidrigen Tat ein ursächlicher und symptomatischer Zusammenhang besteht. Dieser ursächliche Zusammenhang ist gutachtlich darzustellen. Sofern auch die Symptomatizität bejaht werden kann, sind vom Gutachter dann in weiteren Schritten zunächst mögliche Auswirkungen der psychopathologischen Symptome auf die Einsichtsfähigkeit beim Täter zum Tatzeitpunkt zu erörtern. Sofern sich eine störungsbedingte fehlende Fähigkeit zur Einsicht in das Unerlaubte einer Tat nicht begründen lässt, ist in einem nächsten Schritt zu prüfen, ob die Fähigkeit des Täters, seiner Einsicht gemäß zu handeln, zum Tatzeitpunkt störungsbedingt erheblich beeinträchtigt oder aufgehoben war (Steuerungsfähigkeit). Nur wenn vom Gericht festgestellt wird, dass die Voraussetzungen des § 20 StGB oder des § 21 StGB vorliegen, ergibt sich die Notwendigkeit zu Ausführungen im Hinblick auf § 63 StGB (Unterbringung in einem psychiatrischen Krankenhaus), wohingegen eine Unterbringung gemäß § 64 StGB grundsätzlich auch bei erhaltener Schuldfähigkeit in Betracht kommen kann. Für diese Fragestellungen ist die differenzialdiagnostische Unterscheidung zwischen einer primären psychotischen Störung und einer SIPS aber nicht nur von akademischem Interesse. Die Unterbringung in einem psychiatrischen Krankenhaus gemäß § 63 StGB setzt nämlich einerseits voraus, dass eine Wahrscheinlichkeit höheren Grades dafür besteht, dass der Täter zukünftig infolge seines „Zustandes“ erhebliche rechtswidrige Taten begehen wird. Die unterzubringende Person muss eine rechtswidrige Tat begangen haben, die auf einen die Annahme der §§ 20, 21 StGB rechtfertigenden „dauerhaften Defekt“ zurückzuführen ist (BGH 3 StR 79/03). Die rechtlichen Begriffe des „Zustandes“ oder des „dauerhaften Defektes“ sind aber nicht ohne Weiteres auf eine SIPS anzuwenden, da es sich dabei um eine vorübergehende Störung handelt, die sich gemäß den ICD-10-Kriterien innerhalb eines Monats teilweise, innerhalb von 6 Monaten vollständig, zurückbildet. Gutachtliche Dilemmata können sich dabei in vielfältiger Weise ergeben. Eine sorgfältige Anamnese und Diagnostik kann die Differenzialdiagnose meistens ermöglichen, verbleibende Zweifel müssen in einem Gutachten aber offengelegt werden, um Fehleinweisungen im Maßregelvollzug zu vermeiden. Exemplarisch sei folgende Konstellation beschrieben, die in der Praxis zu einer Fehlweinweisung führen kann: Sowohl zum Tatzeitpunkt als auch bei der gutachtlichen Exploration finden sich produktiv psychotische Symptome, es handelt sich um die erste psychotische Episode, Anamnese und Drogenscreening geben Hinweise auf einen langjährigen Cannabis- und Amphetaminkonsum, die Abhängigkeitskriterien sind erfüllt, der letzte Drogenkonsum war vor 4 Monaten. Ohne weitere diagnostische Erkenntnisse können die psychiatrischen Voraussetzungen des § 63 StGB bei diesem Kenntnisstand nicht bejaht werden. Dennoch ist es natürlich möglich, dass der Proband neben der Substanzkonsumstörung eine paranoide Schizophrenie entwickelt hat, wobei diese Diagnose erst im weiteren zeitlichen Verlauf (nach 6‑monatiger Abstinenz von psychotropen Substanzen im Sinne der ICD-10-Klassifikation) und u. U. erst nach der Urteilsverkündung gestellt werden kann. Denkbar – und in der Praxis immer wieder vorkommend – ist also sowohl eine Fehlweinweisung gemäß § 63 StGB – wenn es sich doch um eine SIPS gehandelt hat. Denkbar ist aber auch eine Fehleinweisung in eine Maßregel gemäß § 64 StGB, wenn sich im Verlauf doch eine manifeste Schizophrenie entwickelt. Als weitere Konstellation ist zu bedenken, dass es bei fortgesetztem Substanzkonsum auch rezidivierende SIPS geben kann. Abhängig von der Häufigkeit und Frequenz solcher SIPS kommt gemäß der Rechtsprechung in solchen Fällen auch eine Unterbringung nach § 63 StGB in Betracht. Bei einer rasch oder häufig rezidivierenden SIPS kann u. U. nämlich auch die erforderliche Dauerhaftigkeit einer solchen Störung angenommen werden (siehe Konversionsraten [7–9] und forensische Kasuistik). Es gibt diesbezüglich aber keine rechtlich verbindlichen Vorgaben, wie häufig oder wie rasch eine SIPS rezidivieren muss, um den rechtlichen Begriff des „Zustandes“ zu erfüllen. In einem forensischen Gutachten sollte dieser Aspekt im Sinne einer Einzelfallanalyse diskutiert werden. Es ist wichtig, darauf hinzuweisen, dass man sich mit diesen Überlegungen in einem Grenzbereich der Anwendbarkeit des § 63 StGB bewegt. Auf den Primat der juristischen Würdigung sollte bei dieser normativen Entscheidung vom Gutachter dezidiert hingewiesen werden.

### Strukturiertes Vorgehen bei der korrekten Diagnosestellung

Aus den beiden Fallvignetten und der aktuellen wissenschaftlichen Evidenz ergeben sich die folgenden vier strategischen Schritte^2^ für die differenzialdiagnostische Unterscheidung zwischen SIPS und primären Psychosen (z. B. Schizophrenie) (für eine Übersicht s. Tab. 1):

- **Schritt 1 – Früherkennung: **SIPS sind häufig und sollten immer als eine mögliche Differenzialdiagnose psychotischer Syndrome (insbesondere bei wiederholten psychotischen Episoden bei komorbidem Substanzkonsum) in Erwägung gezogen werden. SIPS haben eine niedrige prospektive diagnostische Stabilität mit häufiger Konversion in manifeste Schizophrenie [61]. Die differenzialdiagnostische Abgrenzung der SIPS („psychotische Störung“ nach ICD-10, innerhalb des Kapitels F1) von den Schizophrenien (F20) erfolgt durch folgende Merkmale [62]: (1) Auftreten während oder unmittelbar nach Substanzgebrauch (innerhalb von 48 h), (2) zumindest teilweiser Rückgang innerhalb eines Monats und (3) vollständige Rückbildung innerhalb von 6 Monaten. Das bedeutet, dass nur bei gesicherter Abstinenz von psychotropen Substanzen (v. a. Cannabis und Kokain) und Persistenz der psychotischen Symptome über 6 Monate, bei ursprünglicher mit eindeutiger substanzinduzierten Auslösung der psychotischen Symptomatik, die Diagnose von F1x.5 in F20.x (Störung aus dem schizophrenen Formenkreis) geändert werden soll (s. auch S3-Leitlinie Methamphetamin-bezogene Störungen). Eine differenzialdiagnostische Unterscheidung zwischen SIPS und primären psychotischen Störungen ausschließlich anhand klinischer Symptomatik ist nach gegenwärtiger Datenlage nicht möglich.
- **Schritt 2 – Erfassung der demographischen Daten:** Neben den laborchemischen können auch demographische, klinische, psychometrische und neuropsychologische Befunde Hinweise auf das Vorliegen einer SIPS liefern. Anhand der beiden Fallvignetten und der selektiven Literaturübersicht konnte gezeigt werden, dass Patienten mit SIPS andere demographische Merkmale aufweisen als Patienten mit primären psychotischen Störungen. Strukturierte Interviews inklusive der Informationen über die Lebensgeschichte inklusive der demographischen Daten sind deshalb sowohl für die allgemeinpsychiatrische als auch für die forensische Praxis von großer Relevanz.
- **Schritt 3 – Erfassung der Abstinenzzeit (seit dem letzten Konsum): **Detaillierte Beschreibung der sich präsentierenden Symptome und die zeitliche Beziehung zwischen dem aktuellen Substanzkonsum und den psychotischen Symptomen. Die Informationen aus diesen Quellen sollen kombiniert werden, damit der klinisch tätige Psychiater, Psychologe oder Gutachter den Zeitpunkt und den Verlauf des Substanzkonsums und der psychotischen Symptome rekonstruieren kann. Zu den häufigsten Gründen für Unsicherheiten bezüglich der Länge der Abstinenzzeit gehören vor allem Inkonsistenzen der subjektiven Angaben bezüglich des vorausgegangenen Substanzkonsums und ein oft durch die psychotische Störung bedingtes reduziertes Erinnerungsvermögen. Die Abklärung der Abstinenzzeit kann deshalb durch die Verwendung folgender Informationsquellen verbessert werden: (1) Fremdanamnese durch Familie oder andere nahestehende Personen (ggf. Vernehmung der Familie oder nahestehender Personen in der Hauptverhandlung unter Beachtung eines eventuellen Zeugnisverweigerungsrechts), (2) Durchsicht von Krankenakten (nach schriftlicher Entbindung von der Schweigepflicht) und kritische Bewertung früherer Diagnosen und (3) Sammlung objektiver Indikatoren für den Substanzkonsum, d. h. chemisch-toxikologische Untersuchungen von Urin- und Blutproben, ggf. laborimmunologische Untersuchungen (Speicheltests oder Urin) zwecks semiquantitativer Ergebnisse, Intoxikationszeichen (Cave: massive Intoxikation bei verschluckten Drogenpäckchen [„bodypacking“]; [63–65]), kardiale Symptome (z. B. Tachykardie, EKG-Veränderungen, Myokarditis), Hautexkorationen, abgekaute Backenzähne, lückenhafter, kariöser Zahnstatus (inkl. Mundsoor [„meth-mouth“] bei verminderter Schleimhautsekretion [66]), Kachexiezeichen, Nasennebenhöhlenentzündungen, Schleimhautblutungen, Anosmie, perforierte Nasenscheidewand, sexuell übertragbare Krankheiten (Gonorrhö, Hepatitis[-B/C]- oder HIV-Infektion) sowie physische Traumazeichen (Z. n. Sturz oder Selbstverletzungen). Nicht selten sind auch Genitalverletzungen (z. B. Penisfraktur durch Ruptur des Corpus cavernosum, penetrierende Verletzungen durch Biss, stumpfe Hodenverletzungen) durch riskante sexuelle Praktiken unter Substanzeinfluss (u. a. bei sog. „Chemsex“ [67–69]). Das Drogenscreening untersucht, ob die vorliegende Symptomatik mit psychotropen Substanzen (z. B. Cannabis, Amphetamine, Ecstasy, Kokain, Heroin) assoziiert ist. Eine Vielzahl der sog. „neuen psychoaktiven Substanzen“ (NPS; synthetische Cannabinoide [z. B. „spice“] oder die sog. „research chemicals“, „legal highs“, „herbal highs“) werden mit den gängigen Drogenscreenings allerdings nicht erfasst.
- **Schritt 4 – Erfassung der kognitiven Leistungsfähigkeit:** Eine neuropsychologische Testung kann wichtige Hinweise bezüglich der korrekten Diagnose liefern, weil primäre psychotische Störungen mit Beeinträchtigung der kognitiven Leistungen (reduzierte Konzentration und Aufmerksamkeit, Störungen des Arbeitsgedächtnisses und der Exekutivfunktionen sowie der Verarbeitungsgeschwindigkeit sind für Schizophrenie charakteristisch) assoziiert sind. Patienten mit SIPS können nach Vollremission der psychotischen Symptomatik Normalwerte (wie bei Gesunden) erreichen. Patienten mit SIPS und Cannabiskonsum in der Vorgeschichte zeigen eine bessere kognitive Leistungsfähigkeit als andere psychotische Patienten [70]. Es gibt allerdings auch Hinweise darauf, dass Cannabiskonsum während der Adoleszenz bei Patienten mit einer Schizophrenie mit einer besseren Leistung bei bestimmten kognitiven Aufgaben assoziiert ist [71].

**Table 1:** 

	Substanzinduzierte psychotische Störungen	Primäre psychotische Störungen (v. a. Schizophrenie)
*Inzidenz und Prävalenz*	1,5 bis 6,5 Personen pro 100.000 Einwohner; die Prävalenz liegt bei 20–40 % bei Menschen mit häufigem Substanzkonsum	11 bis 20 Personen pro 100.000 Einwohner; 19 Neuerkrankungen pro 100.000 Einwohner; die Lebenszeitprävalenz liegt zwischen 0,7 % und 1,4 %
*Klinisches Bild*		
Erstmanifestation	Erstmanifestation bei Konsum psychotomimetischer Substanzen in jeder Altersgruppe möglich	Beginn der Erkrankung in der Regel im frühen Erwachsenenalter, bei Frauen später als bei Männern
Entwicklung der charakteristischen Symptome	ICD-10: (1) Auftreten während oder unmittelbar nach Substanzgebrauch (innerhalb von 48 h); (2) zumindest teilweiser Rückgang innerhalb eines Monats und (3) vollständige Rückbildung innerhalb von 6 Monaten DSM 5: (1) Auftreten während oder bis zu 4 Wochen nach Substanzgebrauch, (2) vollständige Rückbildung innerhalb von 4 Wochen	(1) Die Prodromalphase kann Monate oder Jahre dauern (oft ist retrospektiv ein Psychoserisikosyndrom eruierbar), (2) die Symptomremission ist unterschiedlich, abhängig vom Schweregrad, (3) 30–40 % aller Patienten bleiben trotz antipsychotischer Behandlung anhaltend psychotisch
Psychopathologische Symptome	Positiv- und Negativsymptome; häufiger zu beobachten sind vor allem optische Halluzinationen, Größenwahn, Hyperkinese, Sterotypien, Enthemmung, Aggressivität, überreizte Wachheit (v. a. bei amphetaminassoziierten Psychosen)	Positiv- und Negativsymptome (z. B. Anhedonie, Alogie, Abulie, Apathie, sozialer Rückzug); psychomotorische Symptome (z. B. Katatonie)
Typische körperliche Symptome	Kardiale Symptome, Hautexkorationen, abgekaute Backenzähne, lückenhafter, kariöser Zahnstatus (inkl. Mundsoor [„meth-mouth“], Kachexiezeichen, Nasennebenhöhlenentzündungen, Schleimhautblutungen, Anosmie, perforierte Nasenscheidewand, sexuell übertragbare Krankheiten (Gonorrhö, Hepatitis[B/C]- oder HIV-Infektion) sowie physische Traumazeichen (Z. n. Sturz, Selbst- und/oder Genitalverletzungen)	Typisch sind vor allem körperliche Begleiterkrankungen (z. B. Adipositas, Diabetes mellitus, Bluthochdruck, Karies, COPD) im Zusammenwirken mit ungesunden Lebensstilfaktoren
Kognitive Symptome	Akut können alle kognitiven Domänen betroffen sein; rasche (und vollständige) Wiederherstellung der kognitiven Funktionen nach Vollremission der psychotischen Symptomatik ist häufig	Typisch sind reduzierte Konzentration und Aufmerksamkeit sowie Störungen des Arbeitsgedächtnisses, der Exekutivfunktionen und der Verarbeitungsgeschwindigkeit; Interpretation sozialer Situationen, Dekodierung mimischer und prosodischer Veränderungen und Empathie
*Therapie*	Zeitlich begrenzte Gabe atypischer Antipsychotika (z. B. Olanzapin oder Quetiapin) und begleitend Benzodiazepinen; EKG-Monitoring empfohlen; Cave: Wechselwirkungen zwischen psychotomimetischer Substanz (wenn eindeutig bekannt) und Antipsychotika sind zu beachten	Häufig ist eine längerfristige bis lebenslange Therapie (Rückfallprophylaxe) mit Antipsychotika indiziert
*Prognose*	Bei langfristiger Abstinenz sehr gut, vollständige Rückbildung psychotischer und kognitiver Symptome möglich	Die Prognose ist von verschieden biologischen und psychosozialen Faktoren abhängig

*HIV* Humanes Immundefizienz-Virus, *COPD* „chronic obstructive pulmonary disease“

## Fazit für die Praxis

Unterschiede in demographischen, familiären und klinischen Bereichen bekräftigen SIPS und primäre psychotische Störungen als unterschiedliche neurobiologische Entitäten. Eine differenzialdiagnostische Abgrenzung ist wichtig, um eine unbegrenzte (mit Nebenwirkungen assoziierte) antipsychotische Behandlung oder eine Fehleinweisung in die forensische Psychiatrie zu vermeiden.

## Footnotes

1 Diese forensische Fallvignette ist im Internet unter https://www.hrr-strafrecht.de/hrr/5/04/5-540-04.php zu finden.

2 Die hier vorgestellten vier Schritte stellen keinesfalls wissenschaftlich validierte Leitlinien dar. Sie spiegeln vielmehr die aktuelle Datenlage und die klinische Erfahrung der Autoren wider.
